# Comparing insecticide-treated bed net use to *Plasmodium falciparum* infection among schoolchildren living near Lake Victoria, Kenya

**DOI:** 10.1186/s12936-015-1031-6

**Published:** 2015-12-22

**Authors:** Collins Okoyo, Charles Mwandawiro, Jimmy Kihara, Elses Simiyu, Caroline W. Gitonga, Abdisalan M. Noor, Sammy M. Njenga, Robert W. Snow

**Affiliations:** Eastern and Southern Africa Centre of International Parasite Control, Kenya Medical Research Institute, P.O. Box 54840-00200, Nairobi, Kenya; Spatial Health Metrics Group, Kenya Medical Research Institute-Wellcome Trust Research Programme, Nairobi, Kenya; ICF-Measure Programme, Nairobi, Kenya; Nuffield Department of Clinical Medicine, Centre for Tropical Medicine and Global Health, University of Oxford, Oxford, UK

**Keywords:** *Plasmodium falciparum*, Malaria, Long-lasting insecticide-treated net (LLIN), School surveys

## Abstract

**Background:**

Under trial conditions insecticide-treated nets have been shown to provide significant clinical and mortality protection under a range of malaria transmission intensity conditions. There are, however, few operational impact data, notably in very intense transmission conditions. This study, reports on malaria infection among Kenyan schoolchildren living in areas of intense malaria transmission and their reported use of insecticide-treated bed nets.

**Methods:**

5188 children in 54 schools were randomly sampled from seven counties surrounding Lake Victoria between May and June 2014. A questionnaire was administered to schoolchildren in classes 2–6 on the use of a long-lasting, insecticide-treated net (LLIN) the night before the survey and provided a single blood sample for a rapid diagnostic test for malaria infection. Analysis of the impact of insecticide-treated net use on malaria prevalence was undertaken using a multivariable, mixed effects, logistic regression at 95 % confidence interval (CI), taking into account hierarchical nature of the data and results adjusted for school clusters.

**Results:**

The overall prevalence of malaria infection was 48.7 %, two-thirds (67.9 %) of the children reported using LLIN, 91.3 % of the children reported that their households own at least one LLIN and the household LLIN coverage was 2.5 persons per one LLIN. The prevalence of infection showed variation across the counties, with prevalence being highest in Busia (66.9 %) and Homabay (51.8 %) counties, and lowest in Migori County (29.6 %). Generally, malaria parasite prevalence differed between age groups and gender with the highest prevalence occurring in children below 7 years (50.6 %) and males (52.2 %). Adjusting for county and school, there was a significant reduction in odds of malaria infection among the schoolchildren who reported LLIN use the previous night by 14 % (aOR 0.86, 95 % CI 0.74–0.98, P < 0.027).

**Conclusion:**

Malaria transmission continues to be high around Lake Victoria. Despite evidence of increasing pyrethroid resistance and the likely overall efficacy of LLIN distributed several years prior to the survey, LLIN continue to provide protection against infection among school-aged children.

**Electronic supplementary material:**

The online version of this article (doi:10.1186/s12936-015-1031-6) contains supplementary material, which is available to authorized users.

## Background

Malaria continues to pose a significant public health threat across Africa. Achievements in increasing coverage of efficacious interventions have changed the landscape of transmission and disease burdens in some areas but this has not been universal between or within countries [1–3]. This impact diversity remains a challenge to the dogma that ‘one size fits all’ regarding the selection and optimization of control approaches.

Understanding the intensity of parasite transmission in a community is a fundamental prerequisite to the design of malaria control [4]. How transmission intensity changes with increasing coverage of vector control provides a valuable metric of impact, necessary to sustain, increase or revise coverage of intervention tools [5]. The most widely used metric of malaria transmission intensity is parasite prevalence, measured through cross-sectional surveys. There is a growing recognition of the importance of infection prevalence surveys in the design and evaluation of malaria control initiatives in Africa [6]. National household sample surveys that include malaria infection measurements are increasing in number; however, they are expensive, labour-intensive undertakings. It has been proposed that where school attendance is high and malaria transmission is stable, the use of school-based malaria surveys offers a cheaper alternative to examine community-acquired infection prevalence and reported coverage of household vector control [7, 8].

This study reports on a survey of malaria infection among Kenyan schoolchildren living in areas of intense malaria transmission and the relationship between infection in this age group and their reported use of insecticide-treated bed nets (ITN).

## Methods

### Study area and context

The densely populated counties in Western Kenya, which are close to Lake Victoria, have historically supported the most intense, perennial transmission of *Plasmodium falciparum*. By 2009, this region of Kenya continued to represent the majority of Kenya’s hyper-holo-endemic transmission [9] despite significant increases in coverage of long-lasting, insecticide-treated nets (LLIN) through free-mass distribution campaigns in 2006 [9, 10], changes in first-line treatment to artemisinin-based combination therapy [11], and supporting interventions to change community awareness of the threats posed by malaria infection [12]. In addition, paediatric hospital malaria admissions around Lake Victoria had not declined between 1999 and 2009 in contrast to hospitals located elsewhere in Kenya [13]. Confronted with this challenge, the Kenyan Government secured financing from the Global Fund in 2010 to dedicate significant resources to 16 districts (now seven Counties) that occupy the area proximal to Lake Victoria and ensure universal coverage of LLIN combined with indoor residual spraying (IRS) in selected counties. Between May and June 2011, 7.5 million LLIN were distributed during mass campaigns in Nyanza and western regions (formerly known as provinces) and pyrethroid-based IRS was started in Rachuonyo sub-county in Homa Bay County and then extended to Migori and Kisumu Counties. Owing to increasing vector resistance to pyrethroids [14] and procurement anomalies, IRS was stopped in 2013. To compensate for the removal of IRS, mass LLIN distribution campaigns were prioritized in Migori, Homa Bay, Kisumu, Siaya, and Vihiga counties delivering *circa* three million LLIN in October 2014.

Seven counties were selected for the purposes of the current investigation, Bungoma, Busia, Homa Bay, Kakamega, Kisumu, Migori, and Vihiga, all within 50 km of Lake Victoria. These counties have historically been high-transmission districts and form part of the priority county selection for increased control investment by the Kenyan Government. However, Siaya County, nested within the seven counties, was not included in this study since it currently forms part of intensive, community-based malaria investigations [15]. Surveys undertaken among 3733 pupils attending 34 schools in six of the seven selected sub-counties (excluding Migori) sampled between July and November 2009 reported an overall *P. falciparum* infection prevalence of 24 % (range 2–66 %) [16, 17].

### Sampling

Schools were randomly sampled from each county to be able to provide adequate precision (95 % confidence interval, CI) for county level estimates of *P. falciparum* infection prevalence based upon the county predicted infection prevalence in 2009 [18], the number of public primary schools listed by the Ministry of Education per county, and a presumed design effect of clustering between schools of two, derived from previous school surveys in Kenya [16, 19]. At each selected school ten boys and ten girls were randomly selected from classes 2 to 6 to provide *circa* 100 children per school. In schools where the desired sample could not be achieved because of low enrolment, all the students in classes 2–6 were recruited.

### Survey procedures

Trained interviewers asked each participating child about details related to their age, LLIN use the night before the survey, absenteeism from school in the previous 2 weeks, and any illness on the day of the survey. In addition, children were asked to report on the numbers of LLINs in their household. Each child was asked to provide a finger-prick blood sample for a rapid diagnostic test (RDT) (ParaCheck-*Pf* device, Orchid Biomedical Systems, [20]) to detect *P. falciparum*. All children with a malaria-positive RDT were treated with artemether-lumefantrine according to the national malaria treatment guidelines and written advice on subsequent doses provided to the child and class teacher [2].

Information was captured electronically during the interviews using Open Data Kit for android-based smartphones [21] that stored, provisionally checked and later transmitted data to the Kenya Medical Research Institute (KEMRI) headquarters in Nairobi.

All field staff were trained in survey procedures prior to the survey including mock surveys. The teams used in this study had participated in school based health surveys for many years and all were familiar with protocols for sampling and data collection using mobile phone applications. The RDT tests were read according to the manufacturer’s instructions by trained laboratory technologists.

### Ethical considerations

The study protocol received ethical approval from the KEMRI and National Ethics Review Committee (number 2801). Additional approval was provided by the appropriate county-level health and education authorities, who were briefed about the survey. At school level, parental consent was based on passive, opt-out consent rather than written opt-in consent owing to the low risk and routine nature of the study procedures [22]. Individual assent was obtained from each child before participation in the survey.

### Analysis

*Plasmodium falciparum* infection was defined as a positive RDT result and reported use of a net was assumed to be an LLIN as these have replaced untreated nets since 2006 [9, 17, 23]. Based on reported numbers of household occupants and reported number of nets, household universal LLIN coverage was computed as one LLIN for every 1.8 individuals [24]. Proportions were calculated for variables of interest at county-level with a 95 % CI using generalized linear models that accounted for school clusters. Overall, cross-county analysis of the impact of LLIN use on child infection was analyzed, first using univariable analysis allowing for factors associated with malaria infection (gender, class and age group) and described as odds ratios (OR), using mixed effects logistic regression at two levels; pupils were nested within schools selected within counties. To select minimum adequate variables for multivariable analysis, an inclusion criterion of p < 0.1 was pre-specified; however, age was retained as fixed term in the final model regardless of statistical significance due to its known importance. Adjusted OR (aOR) were obtained by mutually adjusting all minimum generated variables using multivariable mixed effects logistic regression at 95 % CI, taking into account hierarchical nature of the data. All analysis was performed using STATA version 12.0 (STATA Corporation, College Station, TX, USA).

## Results

Between May and June 2014, 54 schools were sampled across the seven counties including 5188 children with a mean age of 9.6 years (standard deviation 2.6) and 50.3 % of the children were female. Overall, 48.7 % (95 % CI 45.3–57.3) of the children were positive for *P. falciparum* and 67.9 % (95 % CI 65.7–74.2) reported having slept under an LLIN the night before the survey. Prevalence varied markedly by school (0–2 %) and by county, the county-level infection prevalence was highest in Busia (66.9 %) and Homa Bay (51.8 %), and lowest in Migori (29.6 %) and Bungoma (35 %) counties. Similarly, the reported use of LLINs varied across the counties with the lowest reported use of LLIN being in Kakamega County (51.9 %), and the highest LLIN use by schoolchildren being in Kisumu County (82 %) (Table 1). In terms of household net ownership, 91.3 % (95 % CI 89.2–93.5) of the children reported having at least one LLIN in their households. Three counties (Kisumu, Homabay, Busia) reported overall net ownership of >90 %, whereas Vihiga County reported the least overall net ownership of 85.5 %. Median reported household coverage of LLIN was above 2.3 persons per one LLIN across all sampled counties (Table 1).

**Table 1:** Background characteristics of schoolchildren sampled at 54 schools in seven counties of Western Kenya

County	No. schools	No. pupils examined	Mean age, years (SD)	Infection prevalence (%) [95 % CI]*	Reported LLIN use by child (%) [95 % CI]**	Median household LLIN coverage (IQR)
Bungoma	6	592 (11.4 %)	9.7 (2.5)	212 (35.8) [19.9–64.6]	336 (56.8) [50.0–63.6]	3.0 (2.7)
Busia	11	1097 (21.1 %)	9.8 (2.6)	734 (66.9) [59.8–74.9]	796 (72.6) [64.8–80.3]	2.3 (2.0)
Homa Bay	15	1389 (26.8 %)	9.7 (2.9)	720 (51.8) [41.9–64.2]	1033 (75.2) [67.6–82.7]	2.5 (2.3)
Kakamega	6	585 (11.3 %)	9.3 (2.5)	246 (42.1) [26.5–66.7]	303 (51.9) [42.4–61.3]	3.0 (2.3)
Kisumu	6	621 (12.0 %)	9.7 (2.5)	273 (44.0) [31.7–61.0]	503 (82.0) [77.2–86.8]	2.5 (1.8)
Migori	4	399 (7.7 %)	9.5 (2.4)	118 (29.6) [14.7–59.4]	268 (67.2) [52.6–81.9]	3.0 (3.0)
Vihiga	6	505 (9.7 %)	9.4 (2.6)	225 (44.6) [36.9–53.8]	273 (54.3) [48.4–60.2]	2.5 (2.5)
Total	54	5188	9.6 (2.6)	2528 (48.7) [43.2–54.9]	3512 (67.9) [63.8–72.0]	2.5 (2.3)

* 95 % CIs were obtained using binomial regression model adjusted for school clusters

** 95 % CIs were obtained using generalized linear latent and mixed models (GLLAMM) adjusting for school clusters

Factors associated with *P. falciparum* infection were investigated using univariable and multivariable mixed effects logistic regression reporting OR at 95 % CI and taking into account two-level hierarchy of the data (county and school). The results from univariable analysis are provided as supplementary information (see Additional file 1: Table S1) while those for multivariable model are shown in Table 2. Age was included in the multivariable model regardless of the effect on infection risk. Whereas, children below 7 years were found to be non-significantly (P = 0.066) more likely to have malaria infection compared to older children (aOR 1.20, 95 % CI 0.99–1.45), there was strong evidence of infection risk (P < 0.001) among children in the age group (7–10) years. Male students were significantly (P < 0.001) more likely to be infected compared to female students (aOR 1.39, 95 %CI 1.23–1.58). In the multivariable model, adjusting for clustering between schools and counties, reported LLIN use by the schoolchild was found to significantly (P = 0.027) reduce the risk of malaria infection, (aOR 0.86, 95 % CI 0.74–0.98).

**Table 2:** Factors associated with malaria infection prevalence

Malaria infection	Multivariable logistic	
	aOR (95 % CI)	P value
Age category		
Below 7 years vs above 10 years	1.20 (0.99–1.45)	0.066
(7–10) years vs above 10 years	1.28 (1.11–1.46)	0.000*
Gender		
Male vs female	1.39 (1.23–1.58)	0.000*
Reported ITN use		
Yes vs no	0.86 (0.74–0.98)	0.027*

**aOR** were obtained by mutually adjusting all minimum generated variables using multivariable mixed effects logistic regression at 95 % CI taking into account hierarchical nature

* Significant at P < 0.05

## Discussion

The reported use of LLIN among schoolchildren in Western Kenya in 2014 was high (67.9 %) and considerably higher than previous reports of 33 % LLIN use among schoolchildren in the lakeside region in 2009–2010 [17]. This increase in reported LLIN use is a direct consequence of increased investment in this region of Kenya since 2009 to ensure universal coverage [24] and addresses inequities previously demonstrated in net use within the vulnerable school-age group [25]. Nevertheless, despite high ownership of LLIN, almost half (48.7 %) of the schoolchildren were infected with malaria, twice the levels of infection prevalence described in the same counties in 2009 [17]. It is not clear why infection prevalence has escalated over this period and deserves a more detailed epidemiological and entomological investigation.

It should be noted that the survey occurred before the large mass distribution campaign of LLIN in this area in October 2014. It is not possible to gauge how bio-effective the LLINs were as it was not recorded when the nets were issued; the last mass distribution was in 2011. It is possible that many of the nets reported as being used by the schoolchildren might have been at the end of their useful life [26]. In addition, reduced bio-efficacy to pyrethroids [14], increasing molecular evidence of pyrethroid resistance [27] and possible vector behavioural adaptation [28] have all been recently confirmed in areas close to where the study sampled schoolchildren. Despite these constraints on the impact of LLIN, the study was able to demonstrate a 14 % reduction in the adjusted odds of being infected in this school-aged population living in a high-transmission area of Kenya.

This study’s findings are contrary to previous studies of the impact of treated nets on infection prevalence in this area of Kenya. During one of the pivotal, randomized control trials of ITNs in the 1990s, undertaken in Western Kenya, two cross-sectional surveys among adolescent girls attending 28 schools showed no impact of treated nets on peripheral malaria infection [29]. Analysis of the impact of LLIN on infection prevalence in schoolchildren during national school surveys undertaken between 2009 and 2010 was unable to show a reduced odds of infection associated with LLIN use among schoolchildren in the lakeside region, despite significant reductions in infection associated with LLIN use in the coast and low-transmission highlands [17]. Conversely, in support of this study’s observations, in Vihiga and Kakamega Counties between 2005 and 2007, the adjusted period prevalence of *P. falciparum* infection was significantly reduced among children reported to have slept under treated nets [30].

An interesting observation from this study was that males had a higher significant risk of parasitaemia compared with female counterparts. These sex differences in malaria infection were significant in all counties except Kisumu, Homabay and Bungoma. This observation is consistent with findings from other studies [31], specifically a study in Kakamega County that showed school-aged girls were significantly less likely to be infected than their male counterparts [32]. Although the cause of these sex differences merits further investigation, including the possibility that males produce more attractive chemicals for mosquitoes [32], the production of estrogens by females has been shown to augment antiplasmodial immune responses, whereas testosterone suppresses antiplasmodial immune responses [31].

School surveys provide a rapid means of establishing the use of interventions in the community and the likely impact of these interventions on the risks of malaria infection. They however suffer from the inability to examine the interventions in use in the children’s households and depend on school children’s responses only. In addition, children absent from school on the survey day are not included and these sampling biases might influence estimates of protective effects of ITN. Although such bias is expected to be higher in areas of lower transmission intensity compared to the area selected during the present study.

## Conclusion

This study found a significant association between LLIN use and *P. falciparum* infection in the lakeside zone with overall significant reduction in the adjusted odds of malaria risk of infection. The results from this study therefore have implications for Kenya’s malaria control programme, which focuses on persons with symptomatic *P. falciparum* infection [11]. Future efforts should target getting bed nets to households through school-attending children; this will effectively address LLIN universal coverage and encourage proper usage among schoolchildren.

One major limitation of the present study was the use of a few selected pupils in a few sampled schools and therefore this might not be representative to county population. This study relied on the affirmative answers of the pupils on the use of nets. Finally, this being a cross-sectional study by nature seasonal fluctuations were not reproduced or captured.

## Additional file

10.1186/s12936-015-1031-6Univariable analysis of factors associated with malaria infection prevalence.
