# Supplementary material for: Comparing insecticide-treated bed net use to Plasmodium falciparum infection among schoolchildren living near Lake Victoria, Kenya
Source: Malar J. 2015 Dec 22;14:515. doi: 10.1186/s12936-015-1031-6 (PMC4688986; doi:10.1186/s12936-015-1031-6)
Supplement: Supplementary file 1 — 10.1186/s12936-015-1031-6Univariable analysis of factors associated with malaria infection prevalence. [file 12936_2015_1031_MOESM1_ESM.docx]

**Additional file 1 : Univariable analysis of factors associated with malaria infection prevalence**

| **Malaria infection** | **Univariable logistic regression** | |
| --- | --- | --- |
|  | **OR (95%CI)** | **p-value** |
| Age category |  |  |
| < 7 years vs > 10 years | 1.14 (0.94-1.37) | 0.194 |
| (7-10) years vs > 10 years | 1.21 (1.06-1.39) | 0.004 |
| Gender |  |  |
| Male vs Female | 1.36 (1.21-1.54) | <0.001 |
| Reported ITN use |  |  |
| Yes vs No | 0.85 (0.74-0.98) | 0.023 |
| Reported ITN coverage |  |  |
| Universal vs No coverage | 1.02 (0.88-1.19) | 0.769 |
| Reported ITN ownership |  |  |
| Atleast one ITN vs No ITN | 0.89 (0.71-1.12) | 0.330 |
| Reported number of ITN currently in use |  |  |
| Atleast one ITN vs No ITN | 0.91 (0.75-1.12) | 0.371 |
| Reported fever |  |  |
| Yes vs No | 1.09 (0.96-1.23) | 0.199 |
